# What results to disclose, when, and who decides? Healthcare professionals' views on prenatal chromosomal microarray analysis†

**DOI:** 10.1002/pd.4772

**Published:** 2016-02-17

**Authors:** Shiri Shkedi‐Rafid, Angela Fenwick, Sandi Dheensa, Diana Wellesley, Anneke M. Lucassen

**Affiliations:** ^1^ Clinical Ethics and Law, Faculty of MedicineUniversity of SouthamptonSouthamptonUK; ^2^ Wessex Clinical Genetics ServiceUniversity Hospitals SouthamptonSouthamptonUK

## Abstract

**Objectives:**

This study explored the views of healthcare professionals (HCPs) in the UK about what information should be disclosed, when; and whether women/parents should be given a choice as to what they wish to know.

**Methods:**

Q‐methodology was used to assess the views of 40 HCPs (genetic HCPs, fetal medicine experts, lab‐scientists).

**Results:**

Most participants agreed that variants of unknown clinical significance should not be disclosed. Participants were divided between those who considered variants of uncertain clinical significance helpful for parents and clinicians, and those who considered them harmful. Although recognising the potential disadvantages of disclosing risks for adult‐onset conditions, participants thought it would be difficult to withhold such information once identified. Participants largely supported some parental involvement in determining which results should be returned. Most participants believed that information obtained via CMA testing in pregnancy should either be disclosed during pregnancy, or not at all.

**Conclusion:**

HCPs taking part in the study largely believed that variants that will inform the management of the pregnancy, or are relevant to other family members, should be reported. Recent UK guidelines, published after this research was completed, reflect these opinions. © 2016 The Authors. *Prenatal Diagnosis* published by John Wiley & Sons, Ltd.

## Abstract 2

**What's already known about this topic?**

- Empirical data on healthcare professionals' (HCPs) and parents' experience with prenatal CMA are scarce.
- Published data mainly focused on issues around uncertain results obtained via CMA, and on genetic health professionals.
- The two main difficulties expressed by genetic counsellors associated with testing were interpreting uncertain results, and termination of pregnancies based on uncertain results

**What does this study add?**

- It is the first study examining attitudes of a wide range of professionals involved in CMA testing: laboratory professionals, fetal medicine experts, and genetic health professionals.
- We describe what types of results professionals think should or should not be disclosed and with whom they consider the onus for such decision making should lie.
- These views are reflected in the recent UK guidelines about CMA testing.

## Introduction

Chromosomal microarray analysis (CMA) allows the identification of small gains and losses of genetic material not detected by conventional karyotyping. Because of its increased diagnostic yield, CMA is now the first‐line genetic investigation for individuals with intellectual disability, developmental delay, autistic spectrum disorder, and multiple congenital anomalies.^1^, ^2^ Although CMA has not yet replaced karyotyping in all indications for prenatal diagnosis (such as advanced maternal age or raised risk from biochemical tests), it is fast becoming the recommended test in pregnancies with structural anomalies, and increased nuchal translucency identified on ultrasound scan.^3^, ^4^, ^5^ This higher resolution view of the genome results in a greater chance of revealing: (1) variants of unknown clinical significance (e.g. novel microdeletions/duplications that contain no genes, or genes with no known function), (2) variants with uncertain clinical significance (VOUS) (e.g. microdeletions/duplications which include genes with incomplete penetrance),^5^, ^6^, ^7^, ^8^ (3) predisposition to diseases whose onset is not until adulthood (e.g. deletion of a cancer susceptibility gene),^9^ and (4) findings relevant only to future pregnancies (e.g. a deletion of an x‐linked gene in a female fetus).

The findings generated by CMA raise ethical as well as practical questions. These include: to whom should testing be offered? (e.g. only in pregnancies where ultrasound anomalies are identified, or to all pregnant women?); which findings should be disclosed? (e.g. only those with clear pathogenicity, or also VOUS; childhood‐onset conditions only or also adult‐onset ones?); should women/parents be given a choice as to what type of results they receive?; and how can information about the fetus best be stored for potential future use?

Empirical data on healthcare professionals' (HCPs') experience with prenatal CMA, and stakeholders' views about these questions, are scarce. This is in part because of the, as yet, limited use of this technology in the prenatal setting. Focusing on the disclosure of uncertain results in pregnancy, Bernhardt *et al*.^10^ explored the experiences of genetic counsellors in the United States. They demonstrated that about 40% of respondents were uncomfortable providing counselling regarding VOUS and expressed an interest in additional education regarding VOUS. From a survey distributed to genetic counsellors in the United States and Canada, Mikhaelian *et al*.^11^ found the two main difficulties associated with testing were interpreting uncertain results, and termination of pregnancies based on uncertain results. Using a survey distributed to genetic counsellors in the United States, Walser *et al*. have recently showed that participants found it important to disclose all types of results from prenatal CMA, including risks for adult‐onset conditions (treatable and non‐treatable), carrier status, and VOUS.^12^

Our study set out to explore the views of HCPs about three main aspects of prenatal CMA testing: (1) what information should be disclosed to women/parents, (2) when should it be disclosed (i.e. during pregnancy, or later on in life), and (3) who should decide. Whereas previous studies mainly concentrated on genetic counsellors and on VOUS, we wanted to gain a broader understanding of the issues around such tests, and to include other professionals involved in offering women/parents CMA testing in pregnancy, analysing, interpreting, and communicating the test results.

## Methods

**Figure 1:**
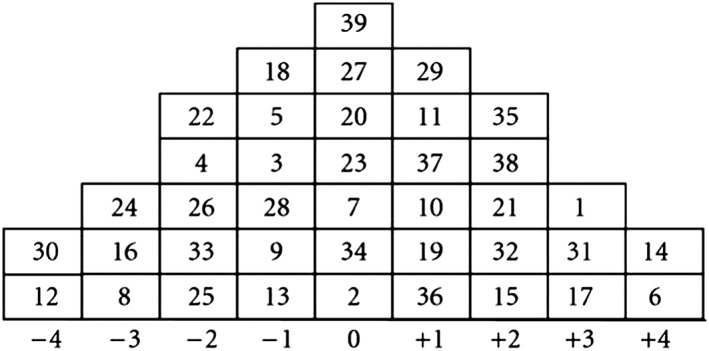
The Q sorting grid and a completed Q‐sort from one of the participants. The numbers in the boxes represent the statements (see Table 2 for the numbered list of statements). In position (+4) are statements that participants most strongly agree with (in this example, statements 14, 6). In position (−4) are statements that participants most strongly disagree with (in this example, statements 30, 12). Position 0 is for statements that participants are unsure, or hold no firm opinions, about. Statements ranked at +1 to +3 are those that participants agree with; those at −1 to −3 are those that they disagree with, or agree with less compared to statements ranked positively

We utilised Q‐methodology, which incorporates both qualitative and quantitative analysis.^13^ Q‐methodology originated in psychology research,^14^ and has since spread to the medical sciences, such as exploring women's and HCPs' views about prenatal testing.^15^, ^16^, ^17^, ^18^ In Q‐methodology, participants are given a set of pre‐determined statements about the research topic, which they are asked to sort (typically on a board), according to their degree of agreement/disagreement with each item (see Figure 1). Instead of indicating the level of agreement/disagreement with each item separately (as is the case with Likert‐style questionnaires), the sorting process asks each participant to prioritise the statements in relation to each other, often resulting in deeper engagement with the research questions. Qualitative data, in the form of participants' oral comments made during the process, are also generated. The completed sorting for each participant (called a Q‐sort) is loaded onto a statistical software program designed for Q‐methodology that looks for groups of participants who have rank ordered the statements in a similar fashion. For instance, if participants A,E,Z had similar rank orders, they would be grouped together. The software then merges together the Q‐sorts of participants in each group to yield a ‘characteristic Q‐sort’ for each group, which serves as an interpretable ‘best‐estimate’ of the way participants in each group have ranked‐ordered the statements; each characteristic Q‐sort then represents a different point of view. The characteristic Q‐sorts (rather than individual Q‐sorts of participants, which contain too many variables for feasible interpretation), together with comments voiced by participants, are used for interpretation.^19^

The study protocol was approved by the University of Southampton, Faculty of Medicine Ethics Committee.

### Development of the statements

We generated a list of statements based on a literature review, two open‐ended interviews with a fetal medicine expert and a consultant in medical genetics, and a focus group with six lab‐scientists, all experienced with prenatal CMA. To clarify the wording of the statements, reduce repetition, and generate new statements, pilot meetings were conducted with four HCPs (three genetic counsellors and a consultant in medical genetics) and two social scientists. The final list consisted of 44 statements (see Table 2), which were printed onto individually numbered cards. Q‐methodology considers between 40 and 80 statements an appropriate number.^19^

### Participants and data collection

At the time of data collection, prenatal CMA testing was carried out in the UK mainly via a research study (EACH),^20^ and as a purely clinical service by very few individual genetic centres, with no consensus or national guidelines. CMA was mainly offered in pregnancies with structural anomalies or increased nuchal translucency. National guidelines were issued well after the completion of the study.^21^ We wanted to explore the views of a range of HCPs and allied HCP (such as lab scientists involved in such testing) with at least some experience in prenatal CMA. We therefore recruited HCPs from centres either taking part in the EACH study, or where prenatal CMA was offered on a clinical basis. As author DGW was part of the EACH study, she could provide the names of these centres as well as of fetal medicine experts familiar with CMA. Participants were recruited in one of three ways: (1) Invitation emails were sent to genetic‐HCPs (gHCPs, i.e. medical geneticists, genetic counsellors, registrars) and to lab scientists who are involved in prenatal testing from the Wessex Clinical Genetics Service, to which authors SSR, DGW and AML belong (altogether 9 of 13 invited gHCPs, and all 4 invited lab scientists agreed to participate). (2) The study was presented to HCPs (mainly gHCPs) attending a national genetics meeting, Genethics Forum,^22^ in February 2014 (29 gHCPs out of a total of 42 attendees). Participants were asked to provide their email address if interested in taking part. Six gHCPs agreed to take part, and meetings were arranged with four of them. (3) Invitation emails were sent to the heads of five genetic laboratories of centres offering prenatal CMA testing, all of whom agreed to participate; and to 17 fetal medicine experts, of whom nine agreed to participate. Participants were asked to provide the contact details of other colleagues who might be interested in taking part, who were then approached (17, of whom 14 agreed to participate). The recommended number of participants in Q‐methodology studies is between 40 and 60 individuals.^19^ Altogether, 45 of the 60 HCPs (75%) who were contacted by email agreed to take part. Two participants cancelled on the day of the meeting, and three did not complete the sorting process and were excluded from analysis. Forty sorting‐configurations from participants belonging to nine centres in England and Scotland were analysed. Participants' professional background, gender, and years of experience are detailed in Table 1. In total, participants included 19 gHCPs, 12 lab scientists, and nine fetal medicine experts.

**Table 1:** Characteristics of participants

Characteristic		Participants n (%)
Professional background	Genetic health professionals (gHCP)	
	Medical geneticist	7 (18)
	Genetic counsellor	9 (23)
	Registrar	3 (8)
	Genetic laboratory scientist	12 (30)
	Fetal medicine experts	
	Fetal medicine midwives	6 (15)
	Fetal medicine consultant	2 (5)
	Obstetrician	1 (2.5)
Gender	Female	32 (80)
	Male	8 (20)
Years of experience	1–5	11 (27.5%)
	6–10	9 (22.5%)
	>10	21 (52.5%)

Data collection took place between November 2013 and April 2014. Meetings lasted on average 60 min and took place mainly at participants' work places. The meetings were audio‐recorded with the consent of participants, and the completed Q‐sort for each participant was noted. Participants' comments were transcribed verbatim.

### Data analysis

The statistical software program PQmethod version 2.35 was used for analysis, as described above.

## Results

Q‐methodology statistical software explored participants' Q‐sorts, and grouped together those who had ranked statements in a similar way. Four groups resulted. See Table 2 for the four characteristic Q‐sorts. Descriptions of each group's viewpoints are presented in Table 3.

**Table 2:** The four characteristic Q‐sorts

Statement	Group1	Group2	Group3	Group4
1. The decision about what information to disclose should be left to healthcare professionals and not involve the parents.	−1	−4	−3	−1
2. Pre‐test discussion with parents about all possible outcomes is practically impossible.	−2	−2	0	3
3. Parents may find pre‐test information about all possible outcomes hard to understand.	1	−1	3	2
4. Parents may perceive aCGH testing as risk‐free, especially if describing it as an add‐on test, and hence may not appreciate in advance its complexity.	1	0	3	3
5. Parents should take an active role in the decision making process about what information they wish to know.	2	2	3	0
6. The lab should report only findings that provide a clinical explanation for the presenting fetal abnormality.	−3	−4	−2	1
7. The lab should report all findings with a known clinical significance (either childhood or adult‐onset)	0	4	−1	0
8. The lab should report all findings with an onset in childhood (even if not relevant to the findings in pregnancy), but not adult‐onset ones.	0	−3	0	1
9. The lab should report findings of unknown clinical significance.^a^	−4	2	−2	−3
10. Clinicians should have a panel of experts that they could consult with should they wish.	3	2	2	4
11. The lab should report findings of uncertain clinical significance (i.e. big range in penetrance).^b^	−2	3	0	−2
12. When the lab thinks that the information is complex, a genetic health professional should be copied in to the report.	4	3	1	4
13. The lab should report all findings, apart from those known to have no clinical significance.	−3	2	−2	0
14. Preferably, it should be fetal medicine experts that discuss aCGH testing with parents.	−1	−3	−1	0
15. Preferably, it should be genetic health professionals that discuss aCGH testing with parents.	1	3	0	2
16. The clinician who receives the report from the lab should decide what information to disclose to parents.	−1	−2	−1	−4
17. Clinicians should tailor the findings which they disclose depending on their assessment of what the parents want to know.	1	−1	0	−3
18. Variants of unknown^a^/uncertain^b^ clinical significance should be discussed by a national panel of experts to decide whether or not they should be disclosed to parents.	1	0	1	1
19. Findings with unknown^a^ medical significance should be disclosed to parents.	−3	−1	−3	−2
20. Medically actionable information, which is only relevant much later on in life, should be disclosed to parents.	0	3	0	1
21. Information about adult‐onset conditions should be disclosed to parents only for conditions with medical intervention (such as cancer predisposition syndromes), but not for those with no medical intervention (such as neurodegenerative disorders).	0	−2	−1	−1
22. Findings with uncertain^b^ medical significance should be disclosed to parents (i.e. big range in penetrance).	−2	1	−2	−2
23. Information that could be relevant to future pregnancies should be disclosed, even if it's not relevant to the present pregnancy (an X‐linked condition, for instance, where the current fetus is a female).	3	4	2	2
24. The decision of what information to disclose should be determined by national guidelines and not left to individual labs/clinicians.	2	1	1	3
25. Each case should be discussed separately. National guidelines may not be applicable to individual cases.	−1	0	1	−1
26. Personal preferences of parents should determine which results are returned, rather than expert opinions of clinicians.	−1	−1	0	−1
27. The possibility of litigation in the future should be taken into consideration whilst deciding what information to disclose to patients.	0	−2	−2	−3
28. Information about adult‐onset conditions should not be disclosed during pregnancy, but after birth.	−2	−3	−3	0
29. Once parents decide not to be told a particular type of information, the decision cannot be changed.	−3	−3	−4	−3
30. Parents are allowed to change their preferences after giving birth and be informed about results that they wished not to know during pregnancy.	−1	0	−1	−1
31. Information about adult‐onset conditions may have an adverse impact on the future child's quality of life.	3	0	2	1
32. Information about adult‐onset conditions may have an adverse impact on parents' interaction with their child.	3	1	2	0
33. Information about findings with uncertain^b^/unknown^a^ clinical significance may lead to parental anxiety.	3	1	4	3
34. One reason why information about variants of unknown^a^/uncertain^b^ clinical significance should not be disclosed to parents is that it may culminate in terminations of healthy pregnancies.	2	−2	1	0
35. Disclosing adult‐onset conditions will remove the child's ability to decide when they are older if they want to be tested, or not.	4	1	4	3
36. Information about adult‐onset conditions may result in discrimination against the future child.	2	0	3	0
37. Prenatal aCGH testing should only be performed on high‐risk pregnancies, but not on low‐risk ones.	2	1	3	2
38. Prenatal aCGH testing should be offered to all pregnant women (even if for economic reasons—some will have to be paid for outside NHS).	0	−3	−4	−3
39. The decision about what information to disclose is no more difficult to make than traditional chromosomal analysis in pregnancy.	−3	−1	−3	−2
40. It is the clinician's duty to disclose unexpected findings with evidence‐based interventions.	0	2	0	2
41. Disclosing incidental findings^c^ is no different from disclosing a chest mass identified through an x‐ray that was carried out to check for pneumonia.	−4	0	−1	−4
42. One reason why incidental findings^c^ should be disclosed is that it gives parents a choice of terminating the pregnancy.	0	0	2	1
43. One reason why variants of unknown^a^/uncertain^b^ clinical significance should be disclosed is that it gives parents a choice of terminating the pregnancy.	−2	−1	−3	−2
44. Incidental findings^c^ should be disclosed to parents if it might benefit other family members (e.g. a deletion of a cancer susceptibility gene).	1	3	1	−1

aCGH, array Comparative‐Genomic‐Hybridization.

a It was explained to participants that findings with unknown clinical significance are novel microdeletions/duplications that contain no known genes, or genes with no known function.

b It was explained to participants that findings with uncertain clinical significance are microdeletions/duplications that contain known genes with incomplete penetrance.

c It was explained to participants that incidental findings are those with known medical significance, but unrelated to the reason for which testing was carried out, either childhood, or adult‐onset.

**Table 3:** Description of the four viewpoints

	Group's central message	Characteristics of group
Group #1	Disclosing for medical benefit	This group consisted of ten participants; three gHCPs, and seven lab scientists. They were:
		• In favour of disclosing findings which could be of medical benefit for the born child's proximate or future health, and for her/his parents/other family members.
		• Against disclosing findings with an unknown, or uncertain medical significance, or with known clinical significance but no management.
Group #2	Disclosing a wide‐range of findings	This group consisted of ten participants; four gHCPs, three lab scientists, and three fetal medicine experts. They were:
		• In favour of disclosing findings with definite or potential clinical significance, for either the present, or future pregnancies; and for both childhood and adult‐onset conditions.
		• In favour of disclosing findings with uncertain clinical significance
		• Against giving parents a choice as to what findings are disclosed
Group #3	Giving parents an active role in deciding what information to receive	This group consisted of ten participants, the majority being gHCPs (eight); one lab‐scientist; and one fetal medicine expert.
		The main issue which distinguished this viewpoint from the others was the emphasis given to parents' role in deciding whether or not they wish to be told of findings with uncertain clinical significance and of adult‐onset conditions.
Group #4	A panel of experts or national guidelines to determine what findings are disclosed	This group consisted of seven participants: three gHCPs; three fetal medicine experts; and one lab scientist. They were:
		• In favor of a panel of experts and national guidelines to determine which findings are disclosed.
		• Not supportive of allowing parents to choose what information they wish to receive.
		• Less supportive of allowing individual clinicians to decide what information to disclose.

We now discuss the issues that were largely agreed upon by participants across all four groups, and those that created the most discussion or argument.

### To disclose or not to disclose, that is the question

It was generally agreed by participants from all four groups that findings of unknown clinical significance should not be included in laboratory reports and should not be disclosed to parents (see statements 9 and 19, Table 2). There was also agreement that information that might be relevant to future pregnancies, even if not relevant to the current pregnancy, should be communicated (statement 23).

As for disclosing VOUS, participants were largely against such disclosure (statement 22). Those who objected to the disclosure of VOUS (mainly those from group one, who generally thought disclosure should be for medical benefit only, and the majority of whom were lab scientists) agreed with the statement that disclosure of VOUS may lead to parental anxiety (statement 33). Another downside of such disclosure is the termination of healthy pregnancies (statements 34). As described by one participant, this is a ‘*lose/lose situation for parents*’ (#25, lab‐scientist), as they either continue the pregnancy with the worry that the VOUS implies pathology, or terminate, unsure whether the fetus was indeed affected.

Participants who supported the disclosure of VOUS voiced a range of reasons for their support: (1) it would be paternalistic not to disclose such findings and to assume that people cannot cope with uncertainty, (2) non‐disclosure could not be justified if a child is born with a condition that is later shown to be associated with the undisclosed information, and (3) when a microdeletion/duplication that might be pathogenic is identified together with a scan abnormality, it could assist women's choices regarding the pregnancy:

‘*given that women can access termination of pregnancy very easily for social reasons, I'm not sure that we should be playing God here and saying you can or can't have a termination when your baby not only has a structural problem, but also a microdeletion, which may have implications for future life*’ (#32, fetal medicine expert).

With regards to disclosing adult‐onset conditions in pregnancy, participants across all groups largely recognised that doing so could have harmful consequences. These included an adverse impact on the quality of life of the child once born and on parents' interaction with their child; removing the child's ability to decide when they are older if and when to be tested; and discrimination against the child (statements 31, 32, 35, 36). Nevertheless, there was generally no objection to disclosing adult‐onset conditions in pregnancy, especially if the conditions were medically actionable albeit not for many years (statements 20, 21, 44). In their comments, participants acknowledged that disclosing medically actionable conditions could benefit the child in two ways: s/he would not miss out on future intervention; and her/his parents could be followed‐up, which in turn also benefits the child, as s/he will not lose a parent to a preventable disease. As for non‐actionable conditions, those who commented in favour of disclosing them thought that receiving the information in pregnancy could give parents a choice to terminate. Participants generally thought that CMA should only be offered to high‐risk pregnancies, and not as a first‐tier test for all women undergoing prenatal diagnosis (statements 37, 38).

### Who decides?

Participants across all groups disagreed with the idea that any decision about what information to disclose should be left to HCPs without parental involvement (statements 1, 5, 16). However, most participants disagreed that personal preferences of parents, rather than expert opinions of clinicians, should determine which results are returned (statement 26). Additionally, participants either agreed that it is a clinician's duty to disclose unexpected findings with evidence‐based interventions, regardless of parental wishes, or were unsure/had no firm opinion about it (statement 40).

Interestingly, participants were divided between those who thought that, with good pre‐test counselling, parents should be able to understand and make informed decisions, and those who believed that even with counselling it would be difficult for parents to understand the complexities around CMA (statements 2–4). For example:

‘*it can be difficult for them [women/parents] to take in a lot of information, but with careful counselling, which is what we all aim to do, I think that most parents are able to take on‐board the subtleties of testing and to make decisions*’ (#10, gHCP)

‘*at the end of the day this is going to be an obstetrician consenting these women, in a busy antenatal clinic, she's going to be really distressed and not listening to a word…you can't possibly go through all the possible outcomes in that session*’ (#31,gHCP)

The majority of participants (especially those belonging to group four, who were a mix of professionals) wanted (inter)national guidance about which findings should be disclosed (statements 18, 24, 25). As expressed by a few participants, a national panel of experts would be considered helpful for facilitating the introduction of CMA into clinical use. They thought that once CMA was being offered as a clinical service to large numbers of pregnant women, such a need would wane with increasing experience. Two practical suggestions were raised by participants to improve the process of prenatal CMA testing: providing women/parents with videos and other multimedia devices prior to the consultation; and educating clinicians involved in prenatal CMA testing.

### When?

Most participants believed that information obtained via CMA testing in pregnancy should either be disclosed during pregnancy, or not at all. If choice was given to parents about what they wish to know, they should be able to change their mind during pregnancy, but not after birth (statements 28–30).

Although there were no statements about the possibility of storing information on fetuses and disclosing it later on in life when it has clinical significance (e.g. adult‐onset conditions), such issues were discussed by many of the participants. Participants generally disagreed with the notion of storing information until the onset of clinical significance, but for different reasons. Some participants cited potential litigation on the grounds of withholding life‐saving information. Others thought parents would lose trust in the doctor–patient relationship. Some participants thought that re‐contacting and disclosing information at a later stage was a good idea in principle, but thought that the practicalities were currently insurmountable:

‘*Apart from anything else, how do you give it [adult‐onset conditions] to them? It's not like you can suddenly re‐contact that family when the child is 18 and say, oh, by the way we found this out when you were in utero and this might be useful for you now*’ (#10, gHCP)

Looking at participants' professional background, two main differences stood out. First, lab scientists were more worried than others about adverse implications of disclosing adult‐onset conditions and VOUS. Second, gHCPs recognised the difficulties in preparing parents for the possible outcomes from prenatal CMA, yet were more likely than other professionals to believe that parents should take an active role in deciding what information to receive.

## Discussion

This study explored the views of HCPs involved in prenatal CMA in the UK, as it moves from being a research technique to being offered to a growing number of pregnant women as part of their clinical care. In deciding which results should be disclosed or not, most participants agreed that variants of unknown clinical significance should not be disclosed, but that findings with proximate clinical significance should be disclosed. However, in between these two extremes, benefit and harm were perceived in different ways by participants regarding the disclosure of VOUS. Bernhardt *et al*.^23^ showed that some women given VOUS continued to worry after delivery and had regrets about having the test, which matches the concerns that some of our participants expressed. Van der steen *et al*.^24^ showed 94% of pregnant couples at increased risk of aneuploidies in pregnancy who opted for invasive prenatal diagnosis, who were given choice between a high and a low‐resolution CMA test, chose testing at high‐resolution, having been informed in advance that this would increase the likelihood of identifying VOUS. Eighty‐four percent of them wished to be told of susceptibility loci for neurodevelopmental disorders (defined to participants as ‘risk factors’; genetic variants that are found in both healthy and affected individuals, with variable expression). When asked about their decision four weeks following results, 90% was satisfied with their choice, but 19% of these were worried about the possible consequences of their decision. Importantly, none of the participants in this study were found to have a susceptibility locus in their fetus. In the recent UK guidelines, it has been recommended that VOUS that cannot be linked to a potential phenotype on the basis of genes involved, and low penetrance neuro‐susceptibility loci, should not be reported.^21^

As for disclosing adult‐onset conditions, although our participants acknowledged the disadvantages of disclosing such findings in pregnancy (for the same reasons that international guidelines almost unanimously recommend against testing in childhood), they also thought that it would be difficult to withhold this type of information once identified. This is also in‐line with the new UK guidelines, according to which variants that inform proximate management of other family members should be disclosed, on the basis of considering the benefit of the child.^21^

Very limited data exist on parent preferences regarding adult‐onset conditions in pregnancy identified incidentally (i.e. not the reason for testing). Srebniak *et al*.^25^ showed that about 55% of parents undergoing prenatal CMA testing wanted to be informed of adult‐onset conditions found incidentally. Kalynchuk *et al*.^26^ and Walser *et al*.^12^ showed that pregnant women and those undergoing prenatal CMA found it important to know about adult‐onset conditions, both treatable and non‐treatable. Nevertheless, the majority of the participants in Kalynchuk *et al.*'s study believed that the identification of an increased risk for adult‐onset and/or a VOUS would cause them anxiety.^26^

Some participants in our study recognised the difficulty of communicating the complexity around potential CMA findings to parents prior to testing. Other studies have demonstrated that participants often do not recall being told, prior to testing, about the possibility of finding VOUS.^23^, ^27^ Van der steen *et al*.^24^ showed that 79% of pregnant couples wished to decide themselves about the resolution of the test and the type of findings they wish to receive. Even if ways are found to assure a satisfactory pre‐test preparation and understanding of potential results, issues remain about how laboratories might filter or blind certain results and/or not record them in patients' records. Although most of our participants thought that parents should be involved to some extent in deciding what information they wished to have, they also thought that national guidelines and consensus about general approaches would be valuable in part because too much choice for parents about multiple different outcomes was considered by many to create a space where informed decision making would be difficult to achieve. To date, various national approaches to prenatal genomic tests have been suggested^28^: The American College of Obstetrics and Gynaecology recently recommended that all findings, with and without known pathogenicity, and regardless of the age of onset, are reported.^29^ Other countries, especially in Europe, support a more restricted disclosure approach, often with the assistance of an advisory committee for complex findings, whilst giving at least some choice to parents to decide what results they wish to receive.^3^, ^25^, ^30^ According to the new UK guidelines, and contrary to the opinion of some of our participants, the only choice given to women/parents is whether or not to have prenatal CMA, and no choice is given regarding which findings are disclosed.^21^

At present, CMA is performed on invasively obtained fetal cells, and mainly in pregnancies with ultrasound abnormalities. Based on the higher detection rates and a shorter turnaround time, it has been suggested that CMA becomes the first‐line test in all women undergoing invasive tests (i.e. replacing karyotyping), for whatever reason.^3^, ^31^, ^32^ Most of the studies exploring experiences of women undergoing CMA to date have been following the identification of abnormal findings on a scan and these are more likely to agree to CMA. How women would react if this was not the case is uncertain. It has been suggested that the risk of miscarriage is the main ‘gatekeeper’ to testing during pregnancy, because once informed of this risk, many women choose not to proceed with testing.^23^, ^27^ Proof of concept whole genome sequencing diagnostics, by means of non‐invasive prenatal testing (NIPT), has already been done although not yet routinely available.^33^, ^34^ Once CMA and other genomic tests can be performed without the risk of a miscarriage, more women/parents might request such tests.^35^ This, together with the spread of genetic medicine to mainstream specialities,^36^ means that more HCPs will be involved in the process of referring women to testing, and in analysing, interpreting and communicating results. Although professional guidelines in Europe and the US stress the importance of pre‐test face‐to‐face genetic counselling, preferably by a gHCPs,^37^ this may be unrealistic when CMA becomes the first‐line test in all pregnancies, and especially if done via non‐invasive tests. An ‘intermediate’ approach has been suggested, in which only complex cases would be referred to a gHCPs to discuss CMA prior to testing.^38^

This study has several limitations: in spite of a fairly high response rate (75%) and an adequate number of participants for the methodology used, HCPs taking part in this study may not represent the entire population of HCPs in the UK. In addition, our results may be prone to respondent bias, as many of the participants were self‐selected. The number of sorting configurations analysed was within, albeit at the lower limit, of the recommended number.

## Conclusion

Much like the recommendations issued recently in the UK, participants taking part in this study largely believed that disclosure of findings obtained via CMA should be based on their present and future clinical consequence for that child, or other family members.

Empirical data are now required on (1) how VOUS and predictions about adult‐onset conditions affect parental choice regarding termination of pregnancy, (2) how such information affects parents and their future child's quality of life and interactions within the family, and (3) how and when adult‐onset conditions are communicated to the child.

## Acknowledgements

We thank the participants for their willingness to participate and share their experiences and views. This study could not have been accomplished without the assistance and input of Gill Crawford from CELS, University of Southampton, and Sara Pervais, from the University of Southampton.

**What's Already Known About This Topic?**

- Empirical data on healthcare professionals' (HCPs) and parents' experience with prenatal CMA are scarce.
- Published data mainly focused on issues around uncertain results obtained via CMA, and on genetic health professionals.
- The two main difficulties expressed by genetic counsellors associated with testing were interpreting uncertain results, and termination of pregnancies based on uncertain results

**What Does This Study Add?**

- It is the first study examining attitudes of a wide range of professionals involved in CMA testing: laboratory professionals, fetal medicine experts and genetic health professionals.
- We describe what types of results professionals think should or should not be disclosed and with whom they consider the onus for such decision making should lie.
- These views are reflected in the recent UK guidelines about CMA testing.
